# Sex differences in the association between green tea consumption and hypertension in elderly Chinese adults

**DOI:** 10.1186/s12877-021-02431-3

**Published:** 2021-09-07

**Authors:** Xiaodong Peng, Mengxia Zhang, Xuesi Wang, Kui Wu, Yukun Li, Linling Li, Jiaxue Yang, Yanfei Ruan, Rong Bai, Changsheng Ma, Nian Liu

**Affiliations:** 1 Department of Cardiology, Beijing Anzhen Hospital, Capital Medical University, Beijing, China; 2 National Clinical Research Center for Cardiovascular Diseases, Beijing, China; 3 Department of Cardiology, Beijing ChuiYangLiu Hospital, Beijing, China

**Keywords:** Sex difference, Green tea, Tea, Blood pressure, Hypertension, Elderly, China

## Abstract

**Background:**

Green tea has been one of the most popular beverages in China since ancient times. Mixed results concerning the effect of green tea consumption on the incidence of hypertension have been published over the past decades. However, no previous studies have focused on longevous individuals in China and the sex differences in the association between habitual green tea intake and hypertension.

**Methods:**

The data extracted from the database of the Chinese Longitudinal Healthy Longevity Survey (CLHLS) in 2018 were used for a secondary analysis. Logistic regression models were employed to examine the odds ratio (OR) of daily green tea consumption on the incidence of hypertension by sex.

**Results:**

A total of 9277 individuals were included in the analysis (39.8% were men). The included individuals had a mean age of 80.9 and 84.8 years for those who drank green tea daily and those who had never, respectively (*p* <  0.001). The incidence of hypertension varied at baseline according to green tea drinking habit and sex. For women who had a habitual green tea intake or had never drunk green tea, the incidence of hypertension was 47.3 and 43.9%, respectively (*p* = 0.241), whereas it was 51.6 and 39.7% for men (*p* <  0.001). After adjusting for potential confounders, a 38% increase in the risk of hypertension was observed in men who consumed green tea daily (OR, 1.38; 95% CI, 1.15–1.67; *p* <  0.001).

**Conclusions:**

Chinese longevous men had a 38% higher risk of developing hypertension when drinking green tea daily. However, no effect of green tea consumption on the incidence of hypertension in women was found. More attention should be paid to the lifestyle of longevous individuals for health promotion, and a sex-specific approach to deliver care for very elderly people is warranted.

## Background

Green tea is one of the most popular beverages in China, and has attracted the attention of elderly Chinese people given the belief that drinking green tea regularly could benefit hypertension. Indeed, some clinical and fundamental studies have indicated that green tea can reduce blood pressure in patients with hypertension. A previous meta-analysis emphasized the hypotensive effect of green tea by demonstrating that it contributed to a decrease in systolic and diastolic blood pressure by 4.81 mmHg and 1.98 mmHg, respectively [1]. Green tea is the most abundant catechin (polyphenol epigallocatechin-3-gallate [EGCG]), which exerts anti-inflammatory and antioxidant effects to reduce elevated blood pressure [2]. However, the results remain controversial as some investigators found no association between green tea consumption and blood pressure [3].

Importantly, sex differences should also be taken into consideration, as the relationship between beverage consumption and the risk of hypertension varies by sex [4]. Over the past decades, it has been proven that females are more vulnerable to several pathological conditions, especially cardiovascular diseases [5]. Moreover, women are also more likely to receive a less effective treatment, leading to a worse clinical outcome [6]. Estrogen is regarded as a protective factor for hypertension, which may partly liberate women from hypertension at an early age [7]. However, the incidence of hypertension is higher in women aged > 60 years than in men, although it is less likely for females to maintain hypertension control [8]. Thus, it is imperative to effectively manage blood pressure using sex-specific approaches.

Therefore, in the current study, we sought to explore the sex difference in the relationship between green tea consumption and hypertension in the Chinese elderly population.

## Methods

### Study population

The information of this cohort study was obtained from the dataset of the seventh wave of the Chinese Longitudinal Healthy Longevity Survey (CLHLS) in 2018. A detailed description of the study design has been reported previously [9]. A total of 15,874 elderly participants from 23 out of 31 provinces in China were enrolled in this survey, and 12,411 of them were interviewed in 2018. Among the samples, the proportion of the population with advanced age (≥ 80 years) was 67.4%. A standard questionnaire was administered by well-trained practitioners to collect items of interest, including sociodemographic, lifestyle, and disease-related information. The CLHLS study was approved by the Biomedical Ethics Committee of the Peking University. All participants provided written informed consent. The study was conducted according to the Strengthening the Reporting of Observational Studies in Epidemiology (STROBE) reporting guidelines [10].

### Data collection

A face-to-face interview was conducted to complete a questionnaire containing various types of information. Demographic data, including age, sex, residence, and marital status, were recorded. The health-related information reported by participants and lifestyle factors were collected, which included self-reported hypertension (systolic blood pressure ≥ 140 mmHg and/or diastolic blood pressure ≥ 90 mmHg), diabetes, dyslipidemia, heart diseases, stroke or cerebrovascular diseases (CVD), smoking status, drinking status, and dietary habits. Subsequently, the database was screened to exclude samples with unavailable data. The inclusion criteria were as follows: (1) advanced age of ≥65 years; (2) reported the frequency of green tea drinking at present and in the past; (3) described the types of tea that they normally drink; and (4) whether they have hypertension or not. Some important information, such as body mass index (BMI) and educational level of participants, which has been previously proven to be associated with hypertension, was ineligible for this study because numerous data derived from the database were invalid [11, 12].

### Specific definition

The frequency of green tea consumption was defined as “daily” or “never”. Based on the content of the questionnaire applied in the CLHLS program, a daily green tea drinker regularly had regularly consumed green tea since they were at least 60 years old without discontinuity (≥ 5 years). The living location was classified as northern and southern China, which was separated by the Qinling Mountains–Huaihe River line. Cities such as Beijing and Tianjin belong to northern China, whereas provinces such as Hunan and Guangdong are considered to be in southern China. Marital status included married individuals and others. The participants who were currently married and living with or separated from their spouse were defined as married, while the others represented those who were divorced, widowed, or never married. Smoking status was also recorded, and two important timelines (the present and when the participants were 60 years old) were set to distinguish the current (smokers at both timelines), the former (not smokers at present), and never smokers (never smoked). These two timelines were applied to identify whether the participants had a daily diet of fresh vegetables, fruits, or salted preserved vegetables. Self-reported physician-diagnosed medical conditions, including hypertension, diabetes, dyslipidemia, heart diseases, stroke, and cerebrovascular diseases were collected.

The mental symptoms of anxiety were measured using the generalized anxiety disorder (GAD-7) form [13]. The GAD-7 form includes seven questions: (1) “Feeling uneasy, worried, and annoyed?”; (2) “Cannot stop worrying?”; (3) “Worry too much about various things?”; (4) “Too nervous to relax?”; (5) “too anxious to sit still?”; (6) “Easily annoyed or irritated?”; and (7) “Feeling like something terrible will happen.” The score of a single symptom varies from 0 (never) to 3 (almost every day), and the total score ranges from 0 to 21. Elevated symptoms of anxiety were defined when the total score was ≥5 [13]. Other covariates, such as number of hours of sleep, having a pension, drinking daily, and regular physical labor, were extracted from the questionnaire.

### Statistical analysis

The baseline characteristics are described according to sex and the frequency of green tea consumption. Continuous data are presented as the mean ± standard deviation (SD), and categorical data are presented as frequency with percentage, which were analyzed with one-way ANOVA and chi-square test, respectively.

To further investigate the association between daily green tea consumption and the incidence of hypertension, multivariate logistic regression was performed to calculate odds ratios (ORs) with 95% confidence intervals (CIs). Three models based on sex differences were used to adjust for the influence of covariates. Age was adjusted in model 1. Urban residence, living location, pension, marital status, smoking status, sleep hours and eating salt-preserved vegetables daily were adjusted in model 2. All covariates, including comorbid diabetes and dyslipidemia were adjusted in model 3. Sex was added to the model for the overall estimation. In logistic regression analysis, all data were presented as categorical variables. Age was classified into < 80 years old and ≥ 80 years old, while sleep hours were separated into < 6, 6–8, and > 8 h.

Subsequently, subgroup analyses were performed to test the potential influence of sociodemographic and health-related factors, including age, residence, living location, marital status, pension, smoking status, sleep hours (divided into < 6 h, 6–8 h, and > 8 h), comorbid diseases (diabetes and dyslipidemia), and dietary habits (eating salt-preserved vegetables daily). The *P*-value for interaction was assessed by likelihood ratio tests, and all statistical analyses were performed using SPSS software (version 19.0; IBM Corporation, Armonk, NY, USA).

## Results

Of the 15,874 participants, 11,509 were included after excluding individuals aged < 65 years, and those with no record of the frequency of tea drinking, types of tea drinking, and history of hypertension. Finally, a total of 9277 participants who had never drank green tea or had drunk green tea daily were enrolled in this study. The baseline characteristics of the enrolled participants are presented in Table 1. For women, the average age was 84.0 years (SD = 12.0) and 87.7 years (SD = 12.1) in those who did and did not drink green tea, respectively. Men were younger than women, with a mean age of 80.9 years (SD = 10.6) for daily green tea drinkers and 84.8 years (SD = 11.1) for those who had never consumed green tea. Approximately 39.0% of women lived in urban areas, while men who never drank green tea usually lived in rural areas (73.0%). The majority of the participants came from southern China (> 70%). Women were more likely to be divorced, widowed, or never married (< 30%), whereas more than 50% of the men were married. The individuals who had never drank green tea tended to perform labor work regularly (73.2% vs. 66.1% for women, *p* <  0.05, and 75.2% vs. 64.7% for men, *p* <  0.001). It seemed that the women were also less wealthy than the men, as the proportion of women with a pension was obviously lower than that of men. Moreover, over 90% of women never smoked, while current smokers were more common in men (38.3% vs. 23.8% for daily green tea drinking and never green tea drinking, respectively, *p* <  0.001). Current alcohol use was more common in men and daily green tea drinkers.

**Table 1:** Baseline characteristics of the participants by sex

Characteristics	Women			Men		
	Green tea daily (*n* = 298)	Green tea never (*n* = 5343)	*P*-value	Green tea daily (*n* = 624)	Green tea never (*n* = 3012)	*P*-value
Age, mean (SD), y	84.0 (12.0)	87.7 (12.1)	< 0.001	80.9 (10.6)	84.8 (11.1)	< 0.001
Urban residence, n (%)	116 (38.9)	2107 (39.4)	0.861	279 (44.7)	812 (27.0)	< 0.001
Area, n (%)						
Northern China	48 (16.1)	1587 (29.7)	< 0.001	149 (23.9)	893 (29.6)	0.004
Southern China	250 (83.9)	3756 (70.3)		475 (76.1)	2119 (70.4)	
Married, n (%)	114 (38.3)	1447 (27.1)	< 0.001	432 (69.2)	1633 (54.2)	< 0.001
Physical labor regularly, n (%)	197 (66.1)	3910 (73.2)	0.008	404 (64.7)	2265 (75.2)	< 0.001
Having pension, n (%)	103 (34.6)	842 (15.8)	< 0.001	288 (46.2)	787 (50.7)	0.040
Smoking status, n (%)						
Never smoker	270 (90.6)	4969 (93.0)	0.205	187 (30.0)	1526 (50.7)	< 0.001
Former smoker	13 (4.4)	201 (3.8)		198 (31.7)	770 (25.6)	
Current smoker	15 (5.0)	173 (3.2)		239 (38.3)	716 (23.8)	
Current drinking, n (%)	32 (10.7)	264 (4.9)	< 0.001	209 (33.5)	629 (20.9)	< 0.001
Sleeping time, mean (SD), h	7.2 (2.4)	7.4 (2.5)	0.170	7.5 (2.0)	7.7 (2.3)	0.076
Anxiety score, n (%)						
0–4	263 (88.3)	4657 (87.2)	0.582	579 (92.8)	2730 (90.6)	0.087
≥ 5	35 (11.7)	686 (12.8)		45 (7.2)	282 (9.4)	
History of comorbidities, n (%)						
Hypertension	141 (47.3)	2343 (43.9)	0.241	322 (51.6)	1197 (39.7)	< 0.001
Diabetes	44 (14.8)	497 (9.3)	0.002	88 (14.1)	249 (8.3)	< 0.001
Dyslipidemia	34 (11.4)	244 (4.6)	< 0.001	49 (7.9)	122 (4.1)	< 0.001
Heart diseases	75 (25.2)	938 (17.6)	0.001	116 (18.6)	456 (15.1)	0.031
Stroke or CVD	40 (13.4)	530 (9.9)	0.051	78 (12.5)	394 (13.1)	0.694
Eating fresh vegetables daily, n (%)	200 (67.1)	3357 (62.8)	0.136	459 (73.6)	1882 (62.5)	< 0.001
Eating salt-preserved vegetables daily, n (%)	47 (15.8)	722 (13.5)	0.269	76 (12.2)	329 (10.9)	0.364
Eating fresh fruits daily, n (%)	74 (24.8)	1157 (21.7)	0.196	168 (26.9)	614 (20.4)	< 0.001

Compared to women who did not drink green tea, those who drank green tea daily were more likely to have comorbid diabetes (14.8% vs. 9.3, *p* <  0.05), dyslipidemia (11.4% vs. 4.6%, *p* <  0.001), and heart diseases (25.2% vs. 17.6%, *p* <  0.05). No significant difference was found in women with regard to the incidence of hypertension, dietary habits, anxiety scores, and sleep hours. The incidence of hypertension was remarkably higher in men who drank green tea daily (51.6% vs. 39.7%, *p* <  0.001). In addition, a history of diabetes (14.1% vs. 8.3%, *p* <  0.001), dyslipidemia (7.9% vs. 4.1%, *p* <  0.001), and heart disease (18.6% vs. 15.1%, *p* <  0.05), and the dietary habits of eating fresh vegetables (73.6% vs. 62.5%, *p* <  0.001) and fruits (26.9% vs. 20.4%, *p* <  0.001) were more prevalent in men.

**Fig. 1:**
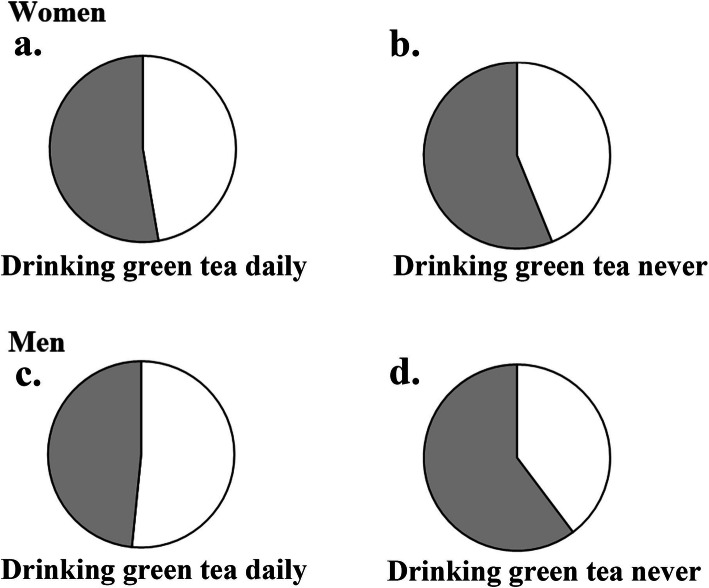
Incidence of hypertension according to green tea drinking habit and sex. The pie charts show the prevalence of hypertension: the white part represents individuals with hypertension while the grey part represents individuals without

Women were not associated with a higher risk of hypertension according to the frequency of green tea consumption (Fig. 1). On the contrary, after adjusting for potential risk factors (model 3), the daily consumption of green tea increased the incidence of hypertension by 38% (95% CI: 1.15–1.67, *p* <  0.001) in men (Table 2). Similarly, the effect of green tea consumption on the incidence of hypertension was detrimental but not significant in the general population (OR, 1.16; 95% CI, 1.00–1.37, *p* = 0.057).

**Table 2:** Association between habitual green tea drinking and risk of hypertension by sex

	Participants, No.	Prevalence of hypertension No. (%)	OR (95% CI)		
			Model 1^a^	Model 2^b^	Model 3^c^
Overall					
Never drinker	8355	3540 (42.5)	Reference	Reference	Reference
Daily drinker	922	463 (50.2)	1.37 (1.19–1.58)^**^	1.20 (1.04–1.39)^*^	1.16 (1.00–1.35)
Women^d^					
Never drinker	5343	2343 (43.9)	Reference	Reference	Reference
Daily drinker	298	141 (47.3)	1.10 (0.87–1.39)	0.93 (0.73–1.18)	0.88 (0.69–1.14)
Men^d^					
Never drinker	3012	1197 (39.7)	Reference	Reference	Reference
Daily drinker	624	322 (51.6)	1.58 (1.33–1.88)^**^	1.43 (1.19–1.71)^**^	1.38 (1.15–1.67)^**^

*OR* Odds ratio. *Statistically significant difference (*p* < 0.05), **statistically significant difference (*p* < 0.001)

^a^Model 1 was adjusted for age and sex

^b^Model 2 was adjusted for Model 1 + urban residence, living location, marital status, pension, smoking status, sleep duration, and eating salt-preserved vegetables daily

^c^Model 3 was adjusted for Model 2 + comorbid diabetes and hyperlipidemia

^d^Model 1–3 used for women and men excluded the sex category

**Fig. 2:**
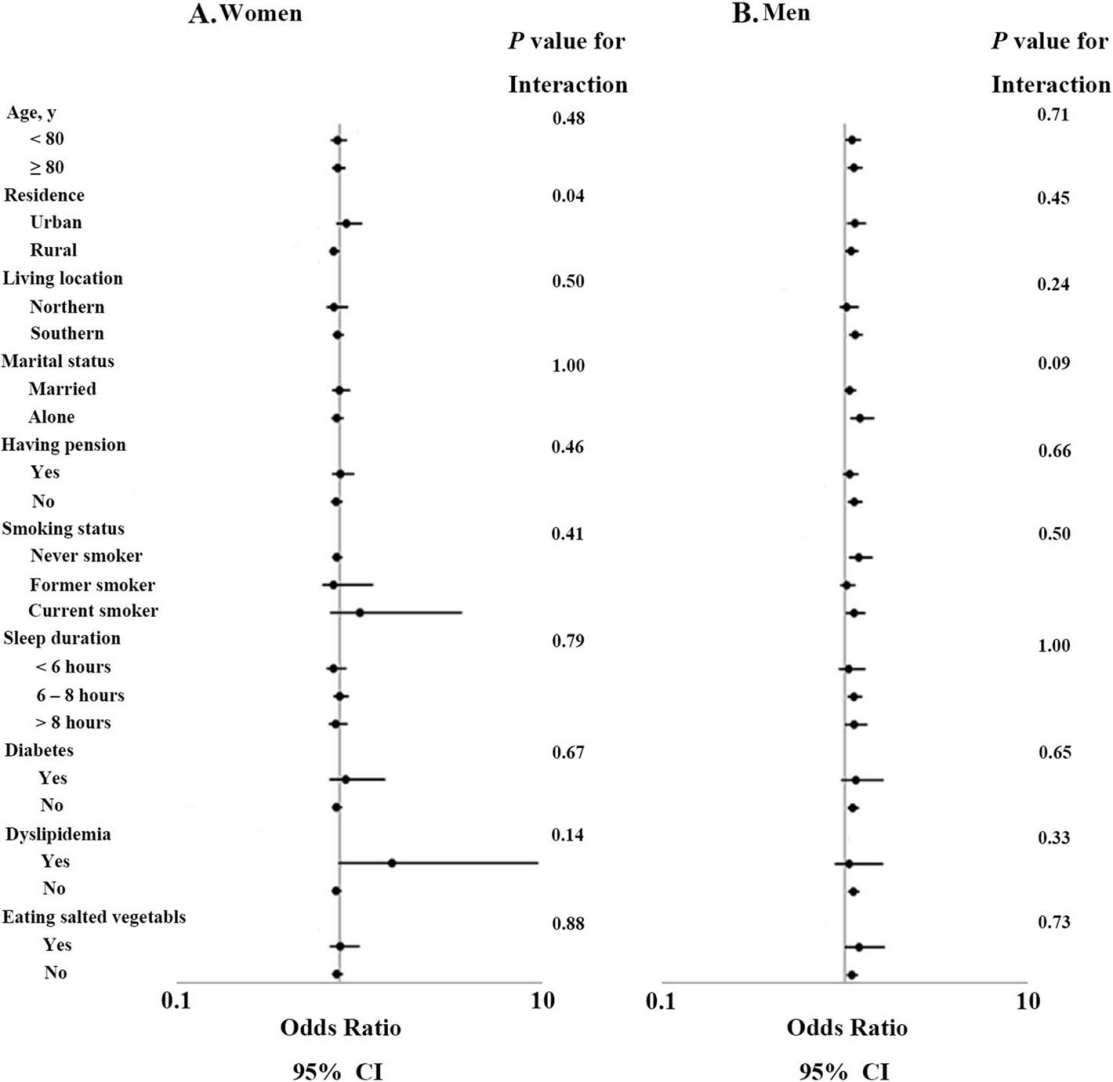
Relation between habitual green tea drinking and risk of hypertension stratified by different factors

The relationship between green tea consumption and hypertension was stratified by potential confounding factors (Fig. 2). For women, those who lived in the urban area seemed to be more vulnerable to hypertension (adjusted OR, 1.30; 95% CI, 0.84–2.02) compared to those in the rural area (adjusted OR, 0.73; 95% CI, 0.53–1.00) (*p* <  0.05). For men, the results did not significantly change after adjusting for age, urban residence, living location, marital status, pension, smoking status, sleep duration, eating salt-preserved vegetables daily, and comorbid diabetes and hyperlipidemia.

## Discussion

In this study, including 9277 individuals of advanced age, men drinking green tea daily (at baseline) were more likely to have comorbid hypertension compared to those who did not drink green tea (51.6% vs. 39.7%). After adjusting for potential confounders, a 38% increase in the risk of developing hypertension among elderly men who enjoyed green tea was estimated. The main finding reflected an interesting fact that green tea consumption may increase 38% the risk of developing hypertension in elderly men from China, whereas no impact of such habits on the incidence of hypertension in women was observed.

The results of studies aiming to investigate the effect of green tea on the risk of hypertension have been mixed. Although a large number of studies have pointed out that it is beneficial for healthy individuals to take green tea as a daily beverage [1, 14, 15], others have found the opposite [3, 16]. One possible explanation is the different compounds that are extracted from green tea, such as caffeine, which is considered to significantly increase the risk of hypertension. A study from Singapore showed that green tea is related to a higher incidence of hypertension, but the risk was attenuated to be non-significant after adjusting for caffeine [16]. In contrast, polyphenols extracted from green tea are believed to be protective in preventing hypertension [17]. Interestingly, different processes of preparing a cup of green tea may lead to a significant difference in the concentrations of caffeine and polyphenols [18]. For example, young tea leaves often contain more caffeine and less polyphenols. Moreover, the biological function of green tea may be stronger when dissolved in 75% ethanol rather than boiling water. Under the condition of higher concentrations of caffeine, tea may have a detrimental impact on the cardiovascular system, contributing to a modest increase in systolic and diastolic blood pressure, bradycardia or tachycardia, and stimulating the release of some neuroendocrine hormones [18]. These dose-dependent effects emphasize the importance of the optimal dosage for green tea intake, which requires further investigation.

Although the hypothesis that green tea could benefit patients with hypertension has been discussed over the past decades, little is known about the effect on very elderly individuals aged ≥80 years. This study found that the overall prevalence of hypertension in the elderly was 43.1%, which was lower than that in adults aged ≥80 years and older (76.5%) [19]. Therefore, it reflected a better state of health of the participants from the CLHLS database, which could represent individuals who are more likely to be longevous.

Notably, a sex difference in habitual green tea drinking and the development of hypertension was observed. Generally, estrogen is regarded as one of the most protective factors for preventing heart diseases and hypertension in women [20]. However, the advantage of sex hormones became negligible among post-menopausal women with a higher testosterone/estradiol ratio, which increases the risk of incident cardiovascular diseases [21]. In this case, the different lifestyles may contribute to the different results between women and men. Most remarkably, women were less likely to be regular alcohol users (5.2% vs. 23.0%) or smokers (3.3% vs. 26.3%) than men. Although alcohol intake and smoking were lower in women than in men, a greater risk of cardiovascular diseases could be found once they started drinking and smoking [22–24]. However, the underlying mechanisms are not well understood, and further sex-specific studies are warranted.

### Strengths and limitations

Two strengths of this study should be emphasized. First, the participants enrolled in this study were from the CLHLS database, which is a well-designed program taking the longevous of age > 80 years as the target population, with the participants distributed throughout 23 provinces in China. Second, there have been limited studies on the effect of green tea drinking on the risk of hypertension in Chinese longevous individuals, and the sex difference in this effect. The major limitations were as follows: (1) the data were extracted from a public database, which was not specifically designed to investigate the topic of interest; therefore, some important information, such as the educational level of participants, may be missing or unavailable; (2) the details of the daily green tea intake and the type of green tea were not designed within the questionnaire, limiting further analysis; and (3) the diagnosis of hypertension was self-reported and no medical records were available.

## Conclusions

Green tea consumption was associated with a 38% increase in the risk of hypertension in Chinese longevous men. However, no effect of green tea consumption on the incidence of hypertension in women was found. More attention should be paid to health promotion to improve the lifestyle of the elderly population in China. Further sex-specific investigations are needed to identify the underlying mechanisms of the observed differences.

## Data Availability

The CLHLS questionnaires are available at https://sites.duke.edu/centerforaging/ programs/chinese-longitudinal-healthy-longevity-surveyclhls/survey-documentation/questionnaires/. The full datasets used in this analysis are available from the corresponding author upon reasonable request.
